# Immune checkpoint inhibitors enhanced the antitumor efficacy of disitamab vedotin for patients with HER2-positive or HER2-low advanced or metastatic gastric cancer: a multicenter real-world study

**DOI:** 10.1186/s12885-023-11735-z

**Published:** 2023-12-15

**Authors:** Caiyun Nie, Weifeng Xu, Yanwei Guo, Xiaohui Gao, Huifang Lv, Beibei Chen, Jianzheng Wang, Yingjun Liu, Jing Zhao, Saiqi Wang, Yunduan He, Xiaobing Chen

**Affiliations:** 1 Department of Medical Oncology, Affiliated Cancer Hospital of Zhengzhou University, Henan Cancer Hospital, No. 127 Dongming Road, Jinshui District, Zhengzhou City, 450008 Henan Province China; 2 State Key Laboratory of Esophageal Cancer Prevention & Treatment, Zhengzhou University, Zhengzhou, 450052 Henan Province China; 3 Henan Engineering Research Center of Precision Therapy of Gastrointestinal Cancer, Zhengzhou, 450008 Henan Province China; 4 Zhengzhou Key Laboratory for Precision Therapy of Gastrointestinal Cancer, Zhengzhou, 450008 Henan Province China; 5 Department of Oncology, Fifth Affiliated Hospital of Zhengzhou University, Zhengzhou, 450052 Henan Province China; 6 Department of Oncology, First Affiliated Hospital of Henan University of Science and Technology, Luoyang, 471003 Henan Province China; 7 Department of General Surgery, Affiliated Cancer Hospital of Zhengzhou University, Henan Cancer Hospital, Zhengzhou, 450008 Henan Province China

**Keywords:** Gastric cancer, HER2, Antibody-drug conjugates drugs, RC48, Immunotherapy

## Abstract

**Background:**

Novel ADC drugs provide a new therapeutic strategy for gastric cancer.The present study aimed to analyze the clinical efficacy and drug toxicities of disitamab vedotin (RC48) plus immune checkpoint inhibitors(ICIs) and RC48 as third-line therapies and beyond for advanced and metastatic gastric cancer patients.

**Methods:**

This was an observational multicenter real-world study.From August 2021 to January 2022,patients with HER2-positive or HER2-low advanced and metastatic gastric cancer and failed from two or more lines of prior therapy were enrolled and treated with RC48 plus ICIs or RC48. In this study, progression free survival(PFS) was the primary end point. Other evaluation indicators were objective response rate(ORR),disease control rate(DCR),overall survival(OS) and drug toxicities.

**Results:**

45 patients were enrolled,of which 25 patients received RC48 plus ICIs,20 patients received RC48.Patients who received RC48 plus ICIs obtained better ORR (36.0% vs. 10.0%, *P* = 0.044) and DCR (80.0% vs. 50.0%, *P* = 0.034) compared with RC48,and simultaneously,the median PFS in RC48 plus ICIs group were superior to RC48 group(6.2 m vs. 3.9 m).The median OS was not reached.No statistically differences were found between HER2-positive and HER2-low group with respect to ORR (27.3% vs. 16.7%, *P* = 0.464),DCR (66.7% vs. 66.7%, *P* = 1.000),median PFS(5.7 m vs. 4.3 m, *P* = 0.299).The most common adverse events (AEs) were decreased white blood count,decreased neutrophil count,fatigue,hypoaesthesia and alopecia.Grade 3–4 AEs occurred in 7(35.0%) patients of RC48 group and 10(40.0%) patients of RC48 plus ICIs group,respectively.

**Conclusion:**

Compared with RC48 monotherapy, ICIs plus RC48 demonstrated superior third-line and beyond therapeutic efficacy for HER2-positive or HER2-low advanced and metastatic gastric cancer patients with manageable safety.

## Introduction

The global cancer statistics data in 2020 showed that the incidence of gastric cancer presents a trend of increasing year by year [1]. It was reported that the global positive rate of HER-2 in gastric cancer is 7.3–20.2%, and the positive rate of HER-2 in Chinese patients is 12–13% [2–3]. Based on the results of phase 3 ToGA study, trastuzumab combined with chemotherapy is the standard first-line treatment manner for HER2-positive advanced and metastatic gastric cancer, and trastuzumab has become the only anti-HER2 targeted drug approved for first-line treatment of advanced and metastatic gastric cancer [4]. However, for patients who have progressed on first-line trastuzumab therapy, there is currently no standard anti-HER-2 regimen.

New antibody-drug conjugate (ADC) drugs, such as T-DXd (DS-8201) and disitamab vedotin (RC48) have shown favorable antitumor efficacy [5–8]. The clinical trial of DESTINY-Gastric01 demonstrated that for patients with HER-2-positive advanced or metastatic gastric cancer who progressed on second-line therapy, DS8201 increased ORR from 14.3 to 51.3%, and PFS from 3.5 months to 5.6 months compared with physician’s choice of chemotherapy (irinotecan or paclitaxel) [9]. RC48 is an innovative ADC drug conjugated with a microtubule inhibitor (monomethyl auristatin E) via a cleavable linker, which directly and potently kills HER2-expressing tumor cells, and at the same time has a bystander effect on heterogeneous tumor cells [10]. RC48 shows potent and safe clinical advantages in various HER2 positive solid tumors such as advanced breast cancer, urothelial carcinoma, gastric cancer and etc [11–13].

There are currently no real-world clinical data on the efficacy of RC48 for gastric cancer. Several clinical studies suggested that immune checkpoint inhibitors (ICIs) may enhanced the antitumor efficacy of RC48. The present study aimed to analyze the clinical efficacy and drug toxicities of RC48 plus immune checkpoint inhibitors(ICIs) and RC48 as third-line therapies and beyond for advanced and metastatic gastric cancer patients in real-world clinical setting.

## Materials and methods

### Patients

From August 2021 to January 2022, patients with HER2-positive or HER2-low advanced and metastatic gastric cancer and failed from two or more lines of prior therapy in 3 institutions were enrolled in this study. Selection criteria: (1) advanced and metastatic gastric cancer; (2) HER-2 positive or HER2-low expression; (3) failed from at least two lines of prior treatment; (4) based on RECIST v1.1, presence of at least one measurable lesion.

The detection of HER2 refers to the Guidelines for HER2 detection in gastric cancer(2016) [14]. HER2 positive was defined as immunohistochemistry (IHC) 3 + or IHC 2+/fluorescence in situ hybridization (FISH) positive and HER2-low was defined as IHC 1 + or IHC 2+/FISH negative. For patients who are diagnosed as advanced and metastatic stage at the initial diagnosis, HER2 is tested on initial biopsy confirmed specimens before treatment. For patients who have relapsed after previous adjuvant therapy, HER2 is detected in metastatic lesion specimens. And HER2 was also evaluated via post-progression new biopsy after two or more lines of prior therapy in a very small number of patients. Anyway, the status of HER2 is based on the latest test results before RC48 treatment.

### Study design and treatment

This was an observational multicenter real-world study, and the patients received RC48 monotherapy or RC48 plus ICIs therapy as third-line therapies and beyond until progressive disease, death or intolerable toxicity occurs. In RC48 monotherapy group, RC48 was administered intravenously, the dosage was 2.5 mg/kg every two weeks. In the RC48 plus ICIs therapy group, RC48 was given at the same dose as RC48 monotherapy. Tislelizumab was given intravenously, the dosage was 200 mg once every three weeks.

### Efficacy and safety assessments

In the RC48 plus ICIs therapy and RC48 monotherapy group, imaging examinations were performed after every 6 weeks. The method of imaging examinations was enhanced computed tomography (CT). According to RECIST version 1.1 response evaluation criteria in solid tumors, the clinical response was divided into complete response (CR), partial response (PR), stable disease (SD), and progressive disease (PD). The ratio of CR plus PR was objective response rate (ORR), and the ratio of CR + PR + SD was disease control rate (DCR). The Common Terminology Criteria for Adverse Events (version 4.0) was used to evaluate drug toxicities.

### Statistical analysis

Difference between groups were compared by Pearson’s chi squared test or Fisher’s exact test. Progression-free survival (PFS) was calculated from using RC48 or RC48 plus ICIs to progressive disease or death of patient. Overall survival (OS) was calculated from using RC48 or RC48 plus ICIs to death of patient or end of follow-up. Kaplan-Meier method and the log-rank test were used to perform survival and prognostic analysis. The follow-up period ends on May 31, 2022. SPSS 22.0 software (SPSS Inc., IL, US) was used for statistical analysis, and *P* < 0.05 was defined as statistically significant.

## Results

### Patient and treatment

45 cases of advanced and metastatic gastric cancer and failed from two or more lines of prior therapy were included. Table 1 summarized the baseline clinicopathological and treatment characteristics of patients. Thirty-three (73.3%) patients in the present study were HER-2 positive, and the other 12 (26.7%) patients had HER2-low expression. All the HER2-positive patients had received trastuzumab targeted therapy in previous first-line therapy. All the patients had received prior therapy, including chemotherapy (45, 100%), antiangiogenic therapy (16, 35.6%) and immunotherapy (26, 57.8%). In the early days, due to the limitation of testing reagents, PD-L1 was not a routine test item in the pathology department of our center. Among the 45 patients in this study, there were 34 patients with PD-L1 expression results, of which 18 were PD-L1 positive and 16 were PD-L1 negative. Twenty five patients received RC48 plus ICIs therapy, and the other 20 patients received RC48 monotherapy. There were no significant differences in the clinicopathological characteristics between RC48 monotherapy and RC48 plus ICIs therapy group.

**Table 1:** Patient and treatment characteristics

Characteristic	All patients n = 45 n (%)	RC48 n = 20 n (%)	RC48 + ICIs n = 25 n (%)	*P*
Age				0.202
Median (range)	62(45–86)	59(45–86)	65(48–82)	
≥ 60	25(55.6)	9(45.0)	16(64.0)	
< 60	20(44.4)	11(55.5)	9(36.0)	
Sex				0.885
Male	31(68.9)	14(70.0)	17(68.0)	
Female	14(31.1)	6(30.0)	8(32.0)	
ECOG				0.438
0–1	34(75.6)	14(70.0)	20(80.0)	
2	11(24.4)	6(30.0)	5(20.0)	
Primary tumor site				0.944
Gastric	29(64.4)	13(65.0)	16(64.0)	
GEJ	16(35.6)	7(35.0)	9(36.0)	
Metastatic site				0.524
Lymph node	31(68.9)	15(75.0)	16(64.0)	
Liver	22(48.9)	9(45.0)	13(52.0)	
Peritoneum	11(24.4)	4(20.0)	7(28.0)	
Lung	12(26.7)	8(40.0)	4(16.0)	
Others	23(51.1)	9(45.0)	14(56.0)	
Number of metastatic sites				0.540
1–2	27(60.0)	11(55.0)	16(64.0)	
≥ 3	18(40.0)	9(45.0)	9(36.0)	
Prior surgery				0.641
Yes	23(51.1)	11(55.0)	12(48.0)	
No	22(48.9)	9(45.0)	13(52.0)	
HER-2 status				0.366
Positive	33(73.3)	16(80.0)	17(68.0)	
HER2-low	12(26.7)	4(20.0)	8(32.0)	
MSI status				0.556
pMMR or MSS	39(86.7)	18(90.0)	21(84.0)	
Unknown	6(13.3)	2(10.0)	4(16.0)	
Previous treatment agents				0.715
Trastuzumab	33(73.3)	16(80.0)	17(68.0)	
Taxane	29(64.4)	14(70.0)	15(60.0)	
Irinotecan	16(35.6)	11(55.0)	5(20.0)	
Platinum	37(82.2)	17(85.0)	20(80.0)	
5-Fluorouracil	39(86.7)	16(80.0)	23(92.0)	
Antiangiogenic agents	16(35.6)	7(35.0)	9(36.0)	
ICIs	26(57.8)	12(60.0)	14(56.0)	

**Abbreviations**: ECOG, Eastern Cooperative Oncology Group performance status; GEJ, Gastroesophageal Junction Tumors; ICIs, immune checkpoint inhibitors

### Treatment response and efficacy

**Fig. 1:**
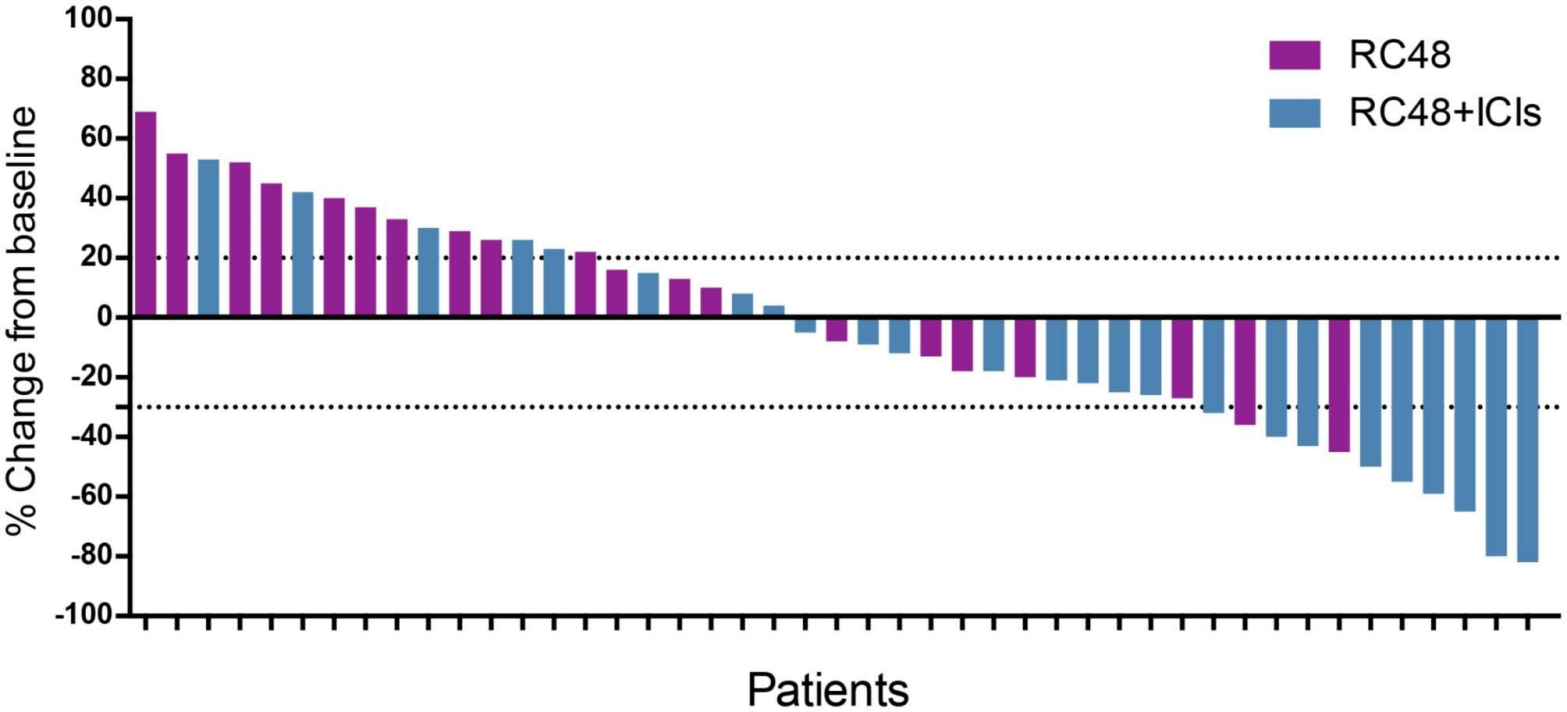
Waterfall plot of the best response change

The overall ORR and DCR in this study were 24.4% (11/45) and 66.7% (30/45), respectively (Fig. 1; Table 2). In the RC48 monotherapy group, the overall ORR and DCR were 10.0% (2/20) and 50.0% (10/20), respectively. In the RC48 plus ICIs therapy group, the overall ORR and DCR were 36.0% (9/25) and 80.0% (20/25), respectively. Patients who received RC48 plus ICIs therapy obtained better ORR (36.0% vs. 10.0%, *P* = 0.044) and DCR (80.0% vs. 50.0%, *P* = 0.034) compared with RC48 monotherapy. No statistically significant difference was found in ORR (27.3% vs. 16.7%, *P* = 0.464) and DCR (66.7% vs. 66.7%, *P* = 1.000) between the HER-2 positive group and HER2-low expression group.

**Table 2:** Efficacy of RC48 in patients with advanced or metastatic gastric cancer

Parameter	Best response				ORR	*P*	DCR	*P*	Median PFS (95%CI)	*P*
	CR	PR	SD	PD						
Total	0	11	19	15	24.4%		66.7%		4.9(2.8-7.0)	
Treatment programs						**0.044**		**0.034**		0.140
RC48	0	2	8	10	10.0%		50.0%		3.9(0.3–7.5)	
RC48 + ICIs	0	9	11	5	36.0%		80.0%		6.2(2.3–10.1)	
HER-2 status						0.464		1.000		0.299
Positive	0	9	13	11	27.3%		66.7%		5.7(3.1–8.3)	
HER2-low	0	2	6	4	16.7%		66.7%		4.3(1.3–7.3)	

**Abbreviations**: CR, complete response; PR, partial response; SD, stable disease; PD, progressive disease; ORR, overall response rate; DCR, disease control rate; PFS, progression free survival; OS, overall survival; ICIs, immune checkpoint inhibitors.

**Fig. 2:**
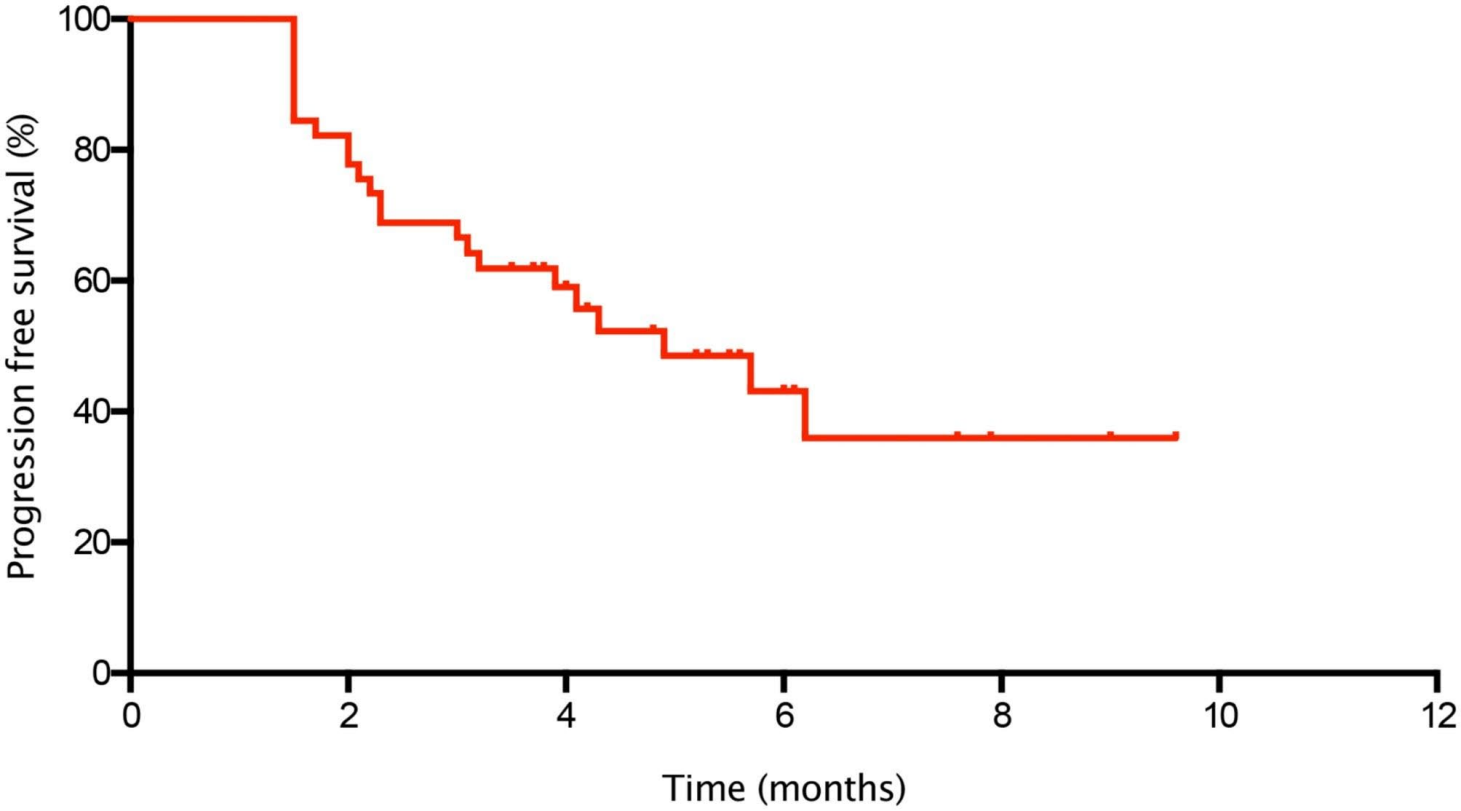
Kaplan-Meier curve of PFS in the general population

**Fig. 3:**
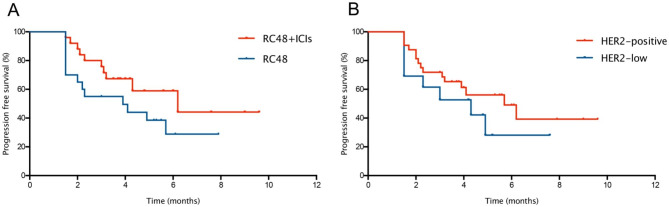
Kaplan-Meier curve of PFS (**A**) in the RC48 plus ICIs and RC48 population. Kaplan-Meier curve of PFS (**B**) in the HER2 positive and HER2-low population

The median follow-up duration was 6.4 m, range from 4.0 to 9.6 m. In the general population, the median PFS was 4.9 months (95% CI = 2.8-7.0) (Fig. 2), and the median OS was not reached. Although the difference was not statistically significant, the median PFS in RC48 plus ICIs therapy group were superior to RC48 monotherapy group (6.2 m vs. 3.9 m, *P* = 0.140, Fig. 3A). No statistically differences were found between HER2-positive and HER2-low group with respect to the median PFS (5.7 m vs. 4.3 m, *P* = 0.299, Fig. 3B).

### Safety

Most of the adverse events (AEs) were grade 1–2 in severity and grade 3–4 AEs occurred in 7 (35.0%) patients of RC48 group and 10 (40.0%) patients of RC48 plus ICIs group, respectively (Table 3). No patients experienced unanticipated toxicities or treatment-related death after treatment. The treatment interruptions as a result of serious adverse events occurred in 6 (30.0%) and 8 (32.0%) patients, respectively. Treatment discontinuation due to adverse events was not observed. Safety data of RC48 were consistent between the two groups. The most common hematologic AEs in RC48 group and RC48 plus ICIs group were decreased white blood count (55.0% vs. 56.0%), decreased neutrophil count (55.0% vs. 52.0%), increased ALT (45.0% vs. 32.0%) and increased AST (45.0% vs. 32.0%). The most common non-hematologic AEs in RC48 group and RC48 plus ICIs group were fatigue (60.0% vs. 56.0%), hypoaesthesia (55.0% vs. 56.0%) and alopecia (65.0% vs. 60.0%).

**Table 3:** Treatment-Related AEs

Adverse Event	RC48			RC48 + ICIs			
	All Grade	≥ Grade3		All Grade	≥ Grade3		
**Hematologic**							
Decreased white blood count	11(55.0)	2(10.0)		14(56.0)		3(12.0)	
Decreased neutrophil count	11(55.0)	2(10.0)		13(52.0)		3(12.0)	
Decreased platelet	4(20.0)	1(5.0)		4(16.0)		0	
Anemia	3(15.0)	1(5.0)		5(20.0)		1(4.0)	
Increased ALT	9(45.0)	0		9(32.0)		1(4.0)	
Increased AST	9(45.0)	0		8(32.0)		1(4.0)	
Hyperbilirubinemia	2(10.0)	0		3(12.0)		0	
TSH elevation	0	0		4(16.0)		0	
**Non-hematologic**							
Fatigue	12(60.0)	0		14(56.0)		0	
Nausea or Vomiting	5(25.0)	0		4(16.0)		0	
Diarrhea	3(15.0)	0		3(12.0)		1(4.0)	
Pyrexia	3(15.0)	0		4(16.0)		0	
Muscle pain/joint pain	4(20.0)	1(5.0)		5(20.0)		0	
Hypoaesthesia	11(55.0)	2(10.0)		14(56.0)		2(8.0)	
Loss of appetite	8(40.0)	0		10(40.0)		0	
Alopecia	13(65.0)	0		15(60.0)		0	
Weight loss	6(30.0)	0		8(32.0)		0	
Rash	2(10.0)	0		4(16.0)		0	
Pruritus	5(25.0)	0		5(20.0)		0	
Pneumonitis	0	0		2(8.0)		1(4.0)	
Hypothyroidism	0	0		4(16.0)		0	
Abdominal pain	3(15.0)	0		2(8.0)		0	

AE, adverse event

## Discussion

The present study evaluated the efficacy and safety of RC48 plus ICIs and RC48 as third-line therapies and beyond for patients with HER2-positive or HER2-low advanced and metastatic gastric cancer. In the general population, ORR and DCR reached 24.4% and 66.7%, and the median PFS reached 4.9 months, which confirmed the well clinical efficacy of this novel ADC drug RC48. In RC48 monotherapy group, ORR and DCR reached 10.0% and 50.0%, and median PFS reached 3.9 months. C008 was a single-arm phase II study, which evaluated the efficacy and safety of RC48 in patients with HER2-overexpressing advanced or metastatic gastric cancer. The ORR and DCR in RC48 therapy group were 24.0% and 42.4%, and median PFS was 4.1 months [12]. Based on this study, RC48 was approved by the National Medical Products Administration (NMPA) as third-line therapies and beyond for HER2 overexpressing locally advanced or metastatic gastric cancer. The efficacy of RC48 in this real-world study was consistent with the results of previous C008 clinical trial.

There is still a lack of effective treatment strategies for the third-line therapies and beyond in gastric cancer, especially for patients with HER2-positive tumors who have failed from first-line trastuzumab therapy. Chemotherapy and immunotherapy are currently the main treatment options. Randomized phase III WJOG 4007 clinical trial evaluated the efficacy of irinotecan and paclitaxel in patients with advanced or metastatic gastric cancer after failure of prior fluoropyrimidine plus platinum chemotherapy. The median PFS of paclitaxel and irinotecan was only 3.6 months and 2.3 months [15]. The phase III randomized controlled ATTRACTION-2 study evaluated nivolumab versus placebo in patients with advanced gastric or gastroesophageal junction (GEJ) adenocarcinoma who progressed after two or lines of chemotherapy. In nivolumab therapy group, the ORR was 11.2% and median PFS was 1.61 months [16]. Clinical KEYNOTE-059 trial evaluated the the safety and efficacy of another ICIs pembrolizumab in patients who had previously received at least 2 lines of treatment. The ORR and DCR were 11.6% and 27.0%, and with a median PFS of 2.0 months [17]. Compared with previous chemotherapy and immunotherapy data, RC48 provides a new therapeutic strategy with superior clinical efficacy.

Our present study confirmed that compared with RC48 monotherapy, patients who received RC48 plus ICIs obtained better ORR and DCR, and simultaneously, the median PFS in RC48 plus ICIs group were superior, which suggests that ICIs enhanced the antitumor efficacy of RC48 and novel ADC drugs combined with immunotherapy may become a new drug combination treatment mode. Basic research has shown that ADC drugs can upregulate the expression of PD-L1 and major histocompatibility complex class I (MHC-I) in tumor cells, which play a synergistic mechanism when used in combination with immunotherapy [18]. Meanwhile, ICIs can promote ADC-induced antitumor immunity [19]. Therefore, ADC drugs combined with immunotherapy may help overcome resistance to either single agent [20–21]. Previous studies of trastuzumab deruxtecan (DS-8201,T-DXd) combined with PD-1 inhibitor in HER2-positive mouse tumor models also found that the anti-tumor effect of this combination therapy mode was better than that of either single drug therapy [22]. Especially in this study, 60.0% of patients in RC48 group and 56.0% of patients in the RC48 plus ICIs group had received prior immunotherapy.

In the RC48 plus ICIs therapy group, 14(56.0%) patients received ICIs in previous treatment scheme, we used ICI again based on the following considerations. The efficacy and outcome of treatment beyond progression (TBP) with ICIs in advanced and metastatic gastric cancer have not been clarified. The ATTRACTION-2 study reported the 3-year update and outcome of TBP with nivolumab in in previously nivolumab treated advanced gastric cancer, which showed that long-term efficacy of nivolumab was confirmed at the 3-year follow-up, and a survival benefit of TBP with nivolumab was suggested [23]. A study conducted in our institute evaluated the efficacy of re-administering ICIs, which included a total of 60 patients. The results showed a median PFS of 2.9 months, ORR of 16.7%, and DCR of 55.0% in the re-administering ICIs group, indicating that re-administering ICIs may be a feasible treatment option for advanced and metastatic gastric cancer. Although we still lack more evidence-based medical evidence to support the re-administering ICIs in previous ICIs treated advanced and metastatic gastric cancer patients, re-administering ICIs is a promising research direction.

The emergence of ADC drugs has expanded the indications of anti-HER2 therapy for advanced or metastatic gastric cancer gastric cancer. On the one hand, after the progression of first-line anti-HER2 therapy for advanced or metastatic gastric cancer, there is no effective anti-HER2 therapy at present. The continuous use of trastuzumab beyond progression (TBP) in gastric cancer is controversial [24–27]. ADC drugs provide an effective back-line anti HER2 treatment strategy. On the other hand, the advent of ADC drugs has redefined the criteria for HER2 classification. Only HER2-positive patients, which was defined as IHC 3 + or IHC 2+/FISH positive, will benefit from traditional anti-HER2 agents. In addition to the efficacy of ADC in this population, it also has promising antitumor activity in patients with HER2 low expression, which was defined as IHC 1 + or IHC 2+/FISH negative [28–29]. Subgroup analysis of the C008 study also showed that both HER2 IHC 3 + and 2 + patients could benefit from RC48 treatment [12]. 26.7% of patients in our present study were HER2 low expression, no statistically differences were found between HER2-positive and HER2-low group with respect to ORR, DCR and median PFS.

The overall safety of RC48 in the present study was consistent with that observed in previous clinical trials [8, 11]. Hematological adverse reactions are the main toxicity of ADC drugs, including decreased white blood count, decreased neutrophil count, anemia and decreased platelet. The mechanism of hematological adverse reactions of ADC drugs has not been fully elucidated. It was generally considered to be related to the drug conjugates, resulting in the bone marrow suppression which was similar to cytotoxic chemotherapy drugs. Due to the lack of clear guidelines or consensus for the management of hematological adverse reactions of ADC drugs, we mostly refer to the guidelines for the management of bone marrow suppression caused by chemotherapy drugs in clinical practice. Compared with RC48 monotherapy, the incidence of adverse reactions in RC48 plus ICIs treatment group did not increase, which means that ICIs will not increase the incidence of RC48 related adverse reactions.

Our study has several strengths and limitations, because it was an observational study with not very large number of patients. Future validation and prospective clinical trials would be needed to confirm the value of ICIs plus RC48 in HER2-positive or HER2-low advanced or metastatic gastric cancer. However, to our knowledge this is the first study exploring novel treatment strategy for advanced or metastatic gastric cancer based on ADC drugs combined with immunotherapy.

## Conclusions

In conclusion, compared with RC48 monotherapy, ICIs plus RC48 demonstrated superior third-line and beyond therapeutic efficacy for HER2-positive or HER2-low advanced and metastatic gastric cancer patients with manageable safety.

## Data Availability

The raw data of this article will be made available by contacting the corresponding author.
